# Comparative Effects of Various Modalities of Cognitive Behavioral Therapy for Insomnia in Adolescents: A Systematic Review and Network Meta‐Analysis

**DOI:** 10.1111/wvn.70158

**Published:** 2026-07-13

**Authors:** Iftitakhur Rohmah, Ti‐No Hsieh, Ya‐Wen Jan, Faizul Hasan, Yi‐Chen Chen, Akhmad Fajri Widodo, Hsin‐Chien Lee, Chia‐Jou Lin, Hsiao‐Yean Chiu

**Affiliations:** ^1^ Faculty of Nursing Chulalongkorn University Bangkok Thailand; ^2^ School of Nursing, College of Nursing Taipei Medical University Taipei Taiwan; ^3^ Department of Nursing National Taiwan University Hospital Taipei Taiwan; ^4^ Department of Psychology Chung Yuan Christian University Taoyuan Taiwan; ^5^ Department of Clinical Psychology Fu‐Jen Catholic University Taipei Taiwan; ^6^ School of Nursing and Midwifery Western Sydney University Penrith Australia; ^7^ Graduate Institute of Injury Prevention and Control, College of Public Health Taipei Medical University Taipei Taiwan; ^8^ Research Center of Sleep Medicine Taipei Medical University Hospital Taipei Taiwan; ^9^ Research Center of Sleep Medicine, College of Medicine Taipei Medical University Taipei Taiwan; ^10^ Graduate Institute of Humanities in Medicine, College of Humanities and Social Science Taipei Medical University Taipei Taiwan; ^11^ Department of Nursing Taipei Medical University Hospital Taipei Taiwan

**Keywords:** adolescents, cognitive behavioral therapy, insomnia, network meta‐analysis, sleep outcomes

## Abstract

**Background:**

Insomnia during adolescence impairs mood and cognitive functioning, yet access to effective behavioral sleep treatment is limited. Although CBTi is considered the first‐line therapy, the relative value of different delivery formats has remained unclear.

**Objective:**

This study systematically evaluated and compared the efficacy of various cognitive behavioral therapy for insomnia (CBTi) delivery formats in improving sleep outcomes among adolescents using a network meta‐analysis (NMA).

**Methods:**

A comprehensive search of five databases, namely PubMed, Embase, PsycINFO, CINAHL, and the Cochrane Library, was conducted through September 28, 2025. Eligible studies were randomized controlled trials (RCTs) involving adolescents with insomnia that examined at least one CBTi modality, including web‐based, face‐to‐face, group‐based, mobile‐app‐based, self‐help, brief CBTi, and sleep hygiene. Both pairwise and network meta‐analyses were conducted using a frequentist random‐effects model. Risk of bias was evaluated using the Cochrane Risk of Bias 2.0 tool, and confidence in evidence was examined using the Confidence in NMA framework. Sleep outcomes included total sleep time (TST), sleep onset latency (SOL), wake after sleep onset (WASO), sleep efficiency (SE), and insomnia severity.

**Results:**

Twenty‐two RCTs evaluating seven CBTi modalities were analyzed. Web‐based CBTi consistently outperformed other delivery formats across most outcomes, significantly improving TST by 33 min, reducing SOL by 23 min, increasing SE by 7%, and reducing insomnia severity by 5 points compared with usual care. P score analysis identified web‐based CBTi as the most effective modality for TST (86%), SOL (79%), SE (74%), and insomnia severity (99%). No significant differences were observed for WASO.

**Linking Evidence to Action:**

Web‐based CBTi is the most effective delivery format for improving sleep outcomes among adolescents. These findings support integrating CBTi into adolescent‐focused sleep care pathways. Implementation of web‐based CBTi as a first‐line option in school and primary‐care pathways could expand access and improve outcomes.

**Trial Registration:**

PROSPERO: CRD420251128688

## 1 Introduction

Insomnia, characterized as difficulty initiating and maintaining sleep or experiencing nonrestorative sleep, is common among adolescents. Insomnia prevalence rates range from 11% to 24% (Johnson et al. 2006; Hysing et al. 2013). Insomnia adversely affects adolescents' academic performance, mental health, and overall quality of life (de Zambotti et al. 2018). Because adolescence is a critical developmental period, effectively addressing insomnia is essential to prevent long‐term adverse outcomes and promote overall health and development.

Cognitive behavioral therapy for insomnia (CBTi) is an effective nonpharmacological intervention for managing insomnia symptoms (Mei et al. 2024; Blake et al. 2017). CBTi typically includes several core therapeutic components, such as sleep restriction, stimulus control, cognitive restructuring, and sleep hygiene education (Trauer et al. 2015). Several CBTi components can be supported through structured sleep education, behavioral counseling, adherence monitoring, and stepped‐care referral pathways, highlighting the relevance of CBTi evidence for nursing, school health, and primary care practice (Haugland et al. 2021; Lunsford‐Avery et al. 2021). CBTi can be delivered in various formats, including group‐based (gCBTi), face‐to‐face (fCBTi), web‐based (wCBTi), mobile‐app‐based (aCBTi), self‐help (sCBTi), and brief CBTi (bCBTi), each designed to enhance accessibility and adherence (de Bruin et al. 2015; Crevits et al. 2024; Bai and Yin 2024; Werner‐Seidler et al. 2023; Chan et al. 2022). Although two conventional pairwise meta‐analyses have reported the effectiveness of CBTi in treating adolescent insomnia (Blake et al. 2017; Mei et al. 2024), comparative evaluations of its different delivery formats remain unavailable. Therefore, identifying the most effective delivery format remains a major challenge for health‐care providers because of the diversity of approaches and varying effects reported in the literature.

Network meta‐analysis (NMA) is a robust analytical approach that integrates direct and indirect evidence across multiple interventions, providing comparative effect and ranking (Lumley 2002; Lu and Ades 2004). Because of the variety and increasing availability of cognitive behavioral therapy delivery methods, an NMA is particularly suited to address the existing gaps in comparative effectiveness research. The optimal CBTi delivery method for improving key sleep parameters, such as total sleep time (TST), sleep onset latency (SOL), wake after sleep onset (WASO), sleep efficiency (SE), and insomnia severity among adolescents remains unknown.

This study systematically evaluated and compared the effects of various CBTi modalities in improving sleep outcomes among adolescents by performing an NMA. The findings of this study provide evidence‐based guidance for clinicians and researchers, facilitating informed decisions regarding optimal CBTi intervention strategies for adolescents experiencing insomnia.

## 2 Methods

### 2.1 Literature Search Strategy

This NMA was performed in accordance with the Preferred Reporting Items for Systematic Reviews and Meta‐Analyses extension for NMA (Hutton et al. 2015). The study protocol was prospectively registered in the International Prospective Register of Systematic Reviews (PROSPERO). Systematic searches were conducted in PubMed, Embase, PsycINFO, CINAHL, and the Cochrane Library from database inception through September 28, 2025. In addition, reference lists of eligible articles were manually screened to identify potentially relevant studies. The search strategies were revised and reformatted line by separating the conceptual axes, including insomnia, CBTi, adolescent population, and randomized trial terms. For PubMed and Embase, native adolescent filters were replaced with explicit controlled‐vocabulary and free‐text population terms. The revised searches identified no additional eligible studies; therefore, the final included studies and quantitative synthesis remained unchanged. The complete search strategies are provided in Table S1.

### 2.2 Selection Criteria

Studies were included if they recruited participants younger than 18 years with either self‐reported insomnia symptoms within recent weeks or months or a formal insomnia diagnosis based on standardized diagnostic criteria (e.g., Diagnostic and Statistical Manual of Mental Disorders, International Classification of Sleep Disorders, International Classification of Diseases, or validated insomnia questionnaires). Eligible studies evaluated at least one CBTi intervention, as defined in Table 1, compared with usual care (UC) or an alternative insomnia intervention, and reported subjective sleep outcomes measured using sleep diaries or validated instruments. Details of the self‐reported insomnia symptom measures used across the included studies are provided in Table S3. Only randomized controlled trials were considered, and no restrictions were imposed on language or publication period. For this review, CBTi was defined as comprising at least four core components: sleep restriction, stimulus control, sleep hygiene (SH), and cognitive restructuring.

**TABLE 1:** Definitions of interventions employed in included trials.

Treatment	Definition
Group cognitive behavior therapy for insomnia (gCBTi)	CBTi delivered in a group setting.
Self‐help cognitive behavior therapy for insomnia (sCBTi)	A CBTi program delivered using self‐guided materials, such as prewritten emails, without individualized therapist support.
Web‐based cognitive behavioral therapy for insomnia (wCBTi)	Web‐based CBTi supported by a real therapist or virtual therapist, involving online supervision and feedback through videoconferencing, algorithms, and email reminders and suggestions.
Mobile‐app‐based cognitive behavioral therapy for insomnia (aCBTi)	CBTi delivered through a mobile application using algorithm‐driven guidance, designed for independent use.
Face‐to‐face cognitive behavioral therapy for insomnia (fCBTi)	Face to face cognitive behavior therapy for insomnia delivered and guided by a sleep therapist.
Brief cognitive behavioral therapy for insomnia (bCBTi)	A condensed version of CBTi, typically delivered in one to four sessions, focusing primarily on core behavioral components such as stimulus control and sleep restriction.
Sleep hygiene (SH)	Education on good sleep habits, such as maintaining a consistent sleep–wake schedule and avoiding caffeine or stimulating activities before bedtime.
Usual care (UC)	The control group received no active intervention and followed standard care practices.

Two independent reviewers (I.R. and A.F.W.) independently screened titles and abstracts to identify potentially eligible studies and removed duplicate records. Full‐text articles were subsequently assessed for eligibility. Any discrepancies between reviewers were resolved through discussion until consensus was reached.

### 2.3 Data Extraction and Risk of Bias Assessment

Data extraction was independently performed by two investigators (I.R. and A.F.W.), who collected information on study characteristics, participant demographics, intervention content, and sleep‐related outcomes. Disagreements were resolved through discussion.

Risk of bias was independently assessed by three reviewers (H.Y.C., I.R., and A.F.W.) using the Cochrane Risk of Bias 2.0 tool (Higgins et al. 2019). This tool evaluates risk across five domains, namely randomization process, deviations from intended interventions, missing outcome data, outcome measurement, and selective reporting. Each domain was rated as low risk, some concerns, or high risk, and an overall judgment was assigned. Any disagreements were resolved through consensus.

### 2.4 Statistical Analysis

All statistical analyses were conducted using R software (version 4.3.3; R Foundation for Statistical Computing, Vienna, Austria). All tests were two‐tailed, and a *p* value of < 0.05 was considered statistically significant. Sleep outcomes, including TST, SOL, WASO, SE, and insomnia severity, were extracted as postintervention means with standard deviations or as mean differences from baseline to endpoint with 95% confidence intervals (CIs).

Pairwise meta‐analyses were first performed using DerSimonian and Laird random‐effects models (DerSimonian and Laird 1986) for direct comparisons of different interventions. Statistical heterogeneity was assessed using Cochrane's Q test (*p* < 0.1 indicated significance) and quantified using the *I* ^2^ statistic, with values exceeding 50% indicating substantial heterogeneity (Higgins et al. 2023).

NMA was conducted using a multivariate random‐effects meta‐regression model within a frequentist framework, integrating both direct and indirect evidence. Treatment effects were summarized using league tables, and intervention rankings were estimated using P scores (ranging from 0 to 1), with higher scores indicating greater effectiveness (Rücker and Schwarzer 2015). Global and local inconsistencies were evaluated using the loop, side‐splitting, and design‐by‐treatment interaction model (Lu and Ades 2004; White 2011). Publication bias was assessed using Egger's test (Chaimani et al. 2013).

Confidence in the network estimates was evaluated using the CINeMA (Confidence in Network Meta‐Analysis) tool based on the GRADE framework (Nikolakopoulou et al. 2020), considering within‐study bias, reporting bias, indirectness, imprecision, heterogeneity, and inconsistency. Each comparison was assigned an overall confidence rating of high, moderate, low, or very low for each comparison (Nikolakopoulou et al. 2020).

### 2.5 Role of the Funding Source

The funders of this study had no role in the study design, data collection, data analysis, data interpretation, or writing of the report. All authors had full access to the study data, and the corresponding author held final responsibility for the decision to submit the manuscript for publication.

## 3 Results

### 3.1 Study Selection, Inclusion, and Characteristics

**FIGURE 1:**
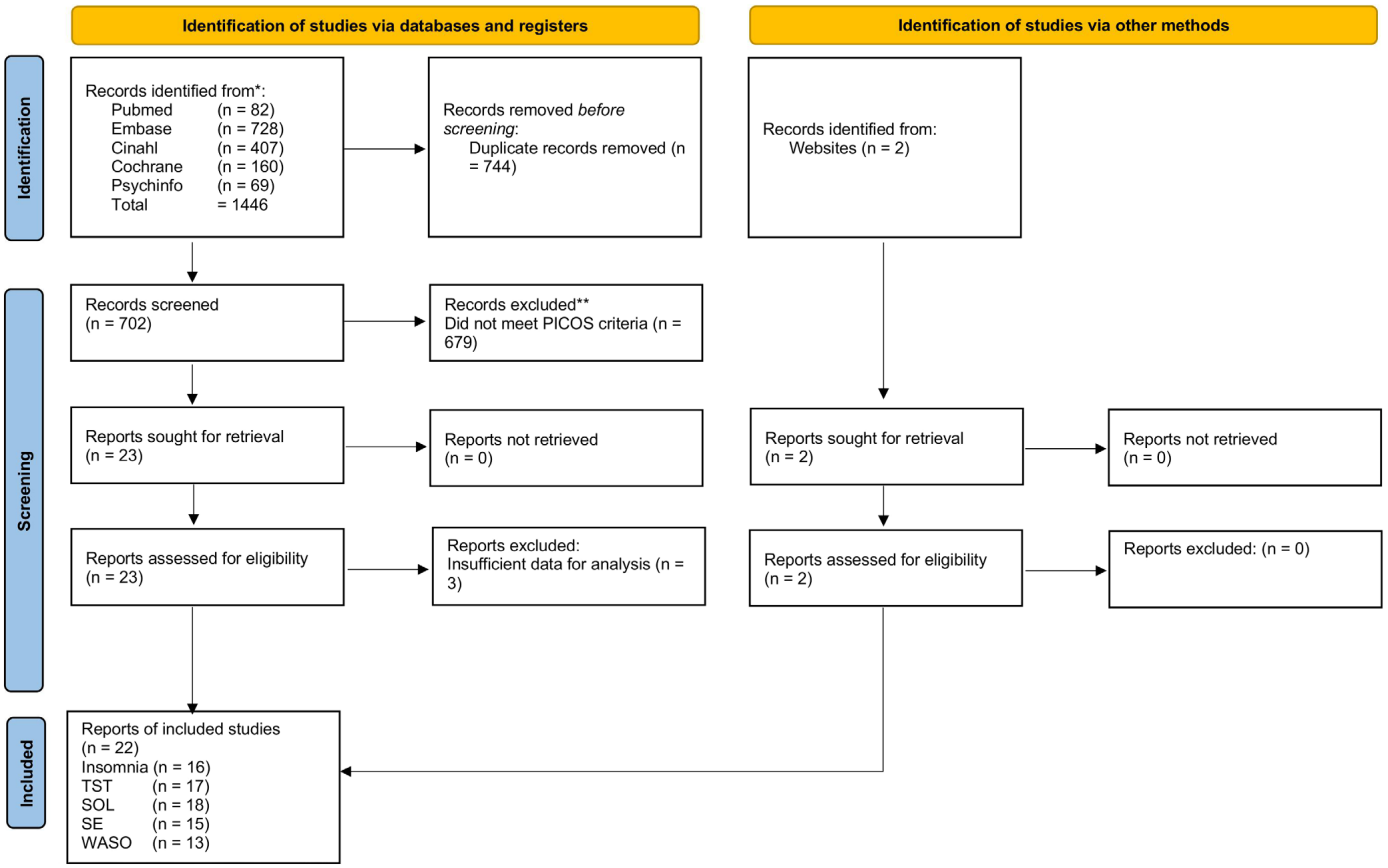
PRISMA 2020 flow diagram. *n*, number of studies; PICOS, patient, intervention, comparison, outcome, and study design; SE, sleep efficiency; SOL, sleep onset latency; TST, total sleep time; WASO, wake after sleep onset.

Figure 1 illustrates the study selection process. After the removal of duplicates and ineligible records, the full text of 23 studies was examined. Of these, three were excluded because their data were insufficient for our analysis, even after contacting the original study authors for additional data (Table S2). Two additional studies were later identified and included, resulting in a final total of 22 studies eligible for the NMA (Table 2).

**TABLE 2:** Study characteristics of included randomized controlled trials.

First author, year	*N*	Treatment/control	CBTi components	Treatment schedule/intensity	Subjective sleep outcomes	Instrument	Dropout (%)
Bai and Yin (2024)	40	wCBTi vs. UC	Core: Cognitive restructuring; Stimulus control; Sleep restriction; Sleep hygiene. Add‐on: Relaxation techniques; Relapse prevention.	30–40 min/session; 1 time/week; 6 weeks; total 180–240 min	TST, SOL, WASO, SE, Insomnia	ISI	20
Blake et al. (2017)	123	gCBTi vs. fCBTi	Core: Cognitive restructuring; Stimulus control; Sleep hygiene. Add‐on: Mindfulness.	90 min/session; 1 time/week; 7 weeks; total 360 min	TST, SOL, WASO	Sleep diary	11.1
de Bruin et al. (2015)	116	wCBTi; gCBTi vs. UC	Core: Cognitive restructuring; Stimulus control; Sleep restriction; Sleep hygiene. Add‐on: Relaxation techniques; Psychoeducation.	90 min/session; 1 time/week; 6 weeks; total 540 min	TST, SOL, WASO, SE	Sleep diary, HSDQ	2.6
de Bruin et al. (2015b)	28	wCBTi vs. UC	Core: Cognitive restructuring; Stimulus control; Sleep restriction; Sleep hygiene. Add‐on: Relaxation techniques; Psychoeducation.	90 min/session; 1 time/week; 6 weeks; total 540 min	TST, SOL, WASO, SE, Insomnia	Sleep diary, HSDQ	0
de Bruin et al. (2018)	116	wCBTi; gCBTi vs. UC	Core: Cognitive restructuring; Stimulus control; Sleep restriction; Sleep hygiene. Add‐on: Relaxation techniques; Psychoeducation.	90 min/session; 1 time/week; 6 weeks; total 540 min	TST, SOL, WASO, SE, Insomnia	HSDQ, Sleep diary	5.1
de Bruin et al. (2014)	26	gCBTi vs. wCBTi	Core: Cognitive restructuring; Stimulus control; Sleep restriction; Sleep hygiene. Add‐on: Relaxation techniques; Psychoeducation.	90 min/session; 1 time/week; 6 weeks; total 540 min	SOL, SE	Sleep diary	3.7
Chan et al. (2021)	205	bCBTi vs. UC	Core: Cognitive restructuring; Stimulus control; Sleep restriction; Sleep hygiene. Add‐on: Relaxation techniques; Psychoeducation.	60 min/session; 1 time/week; 4 weeks; total 240 min	Insomnia, TST	ISI	15
Chan et al. (2022)	73	gCBTi; sCBTi vs. UC	Core: Cognitive restructuring; Stimulus control; Sleep restriction; Sleep hygiene. Add‐on: Relaxation techniques; Psychoeducation; Mindfulness.	60 min/session; 1 time/week; 6 weeks; total 360 min	Insomnia, TST, SOL, SE, WASO	ISI, Sleep diary	6–10
Chen et al. (2024)	242	bCBTi vs. UC	Core: Cognitive restructuring; Stimulus control; Sleep restriction; Sleep hygiene. Add‐on: Relaxation techniques; Psychoeducation.	60 min/session; 1 time/week; 4 weeks; total 240 min	Insomnia	ISI	11.2
Clarke et al. (2015)	41	bCBTi vs. SH	Core: Cognitive restructuring; Stimulus control; Sleep restriction; Sleep hygiene. Add‐on: Relaxation techniques; Psychoeducation.	50 min/session; 1 time/week; 4 weeks; total 200 min	TST, SOL, WASO	Sleep diary	6
Clarke et al. (2016)	212	bCBTi vs. UC	Core: Cognitive restructuring; Stimulus control; Sleep hygiene. Add‐on: Psychoeducation.	50 min/session; 1 time/week; 4 weeks; total 200 min	Insomnia	ISI	5
Crevits et al. (2024)	33	fCBTi vs. UC	Core: Cognitive restructuring; Stimulus control; Sleep restriction; Sleep hygiene. Add‐on: Psychoeducation.	60 min/session; 1 time/week; 5 weeks; total 300 min	TST, SOL, SE, WASO	Sleep diary	16.7
Egbegi et al. (2021)	37	fCBTi vs. UC	Core: Stimulus control; Sleep restriction; Sleep hygiene. Add‐on: Relaxation techniques; Psychoeducation.	45 min/session; 1 time/week; 5 weeks; total 225 min	Insomnia, SOL	ISI	16
Gradisar et al. (2011)	40	fCBTi vs. UC	Core: Cognitive restructuring; Stimulus control; Sleep hygiene. Add‐on: Psychoeducation; Bright light therapy.	60 min/session; 1 time/week; 6 weeks; total 360 min	TST, SOL, WASO	Sleep diary	18
Haugland et al. (2021)	189	fCBTi; bCBTi vs. UC	Core: Cognitive restructuring. Add‐on: Parent involvement.	90 min/session; 1 time/week; 10 weeks; total 900 min	Insomnia, SOL	Sleep diary	9
van der Hoek et al. (2025)	48	wCBTi vs. UC	Core: Cognitive restructuring; Stimulus control; Sleep restriction; Sleep hygiene. Add‐on: Relaxation techniques; Psychoeducation.	30 min/session; 1 time/week; 5 weeks; total 150 min	Insomnia	ISI	10
Moseley and Gradisar (2009)	30	gCBTi vs. UC	Core: Cognitive restructuring; Stimulus control; Sleep hygiene. Add‐on: Psychoeducation; Bright light therapy.	50 min/session; 1 time/week; 4 weeks; total 200 min	TST, SOL	Sleep diary	10
Paine and Gradisar (2011)	42	fCBTi vs. UC	Core: Cognitive restructuring; Sleep restriction; Sleep hygiene. Add‐on: Relaxation techniques; Parent involvement.	45–60 min/session; 1 time/week; 6 weeks; total 270–360 min	SOL, WASO, SE	Sleep diary	2.4
Schlarb et al. (2018)	112	gCBTi vs. UC	Core: Stimulus control; Sleep restriction; Sleep hygiene. Add‐on: Relaxation techniques; Psychoeducation; Parent involvement.	100 min/session; 1 time/week; 6 weeks; total 600 min	SOL, WASO, SE, TST	Sleep diary	15.2
Werner‐Seidler et al. (2023)	147	aCBTi vs. UC	Core: Cognitive restructuring; Stimulus control; Sleep hygiene.	10–15 min/session; 1 time/week; 6 weeks; total 60–90 min	Insomnia	ISI	11
Taylor et al. (2014)	34	fCBTi vs. UC	Core: Cognitive restructuring; Stimulus control; Sleep restriction; Sleep hygiene. Add‐on: Relaxation techniques; Psychoeducation.	50–60 min/session; 1 time/week; 6 weeks; total 300–360 min	SE, SOL, WASO, TST, Insomnia	Sleep diary, ISI	6
Tomfohr‐Madsen et al. (2020)	24	fCBTi vs. UC	Core: Cognitive restructuring; Stimulus control; Sleep restriction; Sleep hygiene. Add‐on: Relaxation techniques; Psychoeducation; Mindfulness; Relapse prevention.	45 min/session; 1 time/week; 6 weeks; total 270 min	Insomnia, SOL, TST, WASO, SE	ISI, Sleep diary	0

*Note: N* indicates the total participants across study arms based on the extracted outcome data.

Abbreviations: aCBTi, app‐based CBTi; bCBTi, brief CBTi; CBTi, cognitive behavioral therapy for insomnia; fCBTi, face‐to‐face CBTi; gCBTi, group CBTi; HSDQ, Holland Sleep Disorder Questionnaire; ISI, Insomnia Severity Index; sCBTi, self‐help CBTi; SE, sleep efficiency; SH, sleep hygiene; SOL, sleep onset latency; TST, total sleep time; UC, usual care; WASO, wake after sleep onset; wCBTi, web‐based CBTi.

Seven intervention types were evaluated across the 22 included studies (Table 1). Table 2 summarizes the characteristics of the included studies, which examined the effects of different CBTi formats. Most trials used a two‐arm parallel design. Intervention durations ranged from 4 to 10 weeks, with one session being conducted per week. Total intervention durations ranged from 150 to 900 min. Common CBTi components included SH, stimulus control, sleep restriction, cognitive restructuring, relaxation techniques, and, in some cases, mindfulness or parental involvement (Trauer et al. 2015; de Bruin et al. 2015). The most frequently used instruments were sleep diaries (*n* = 15) and the Insomnia Severity Index (*n* = 10), followed by the Holland Sleep Disorder Questionnaire (*n* = 3). Dropout rates varied from 0% to 20%. Sleep outcomes were measured consistently, with most studies using both sleep diaries and validated questionnaires to evaluate intervention efficacy.

### 3.2 Network Plots

**FIGURE 2:**
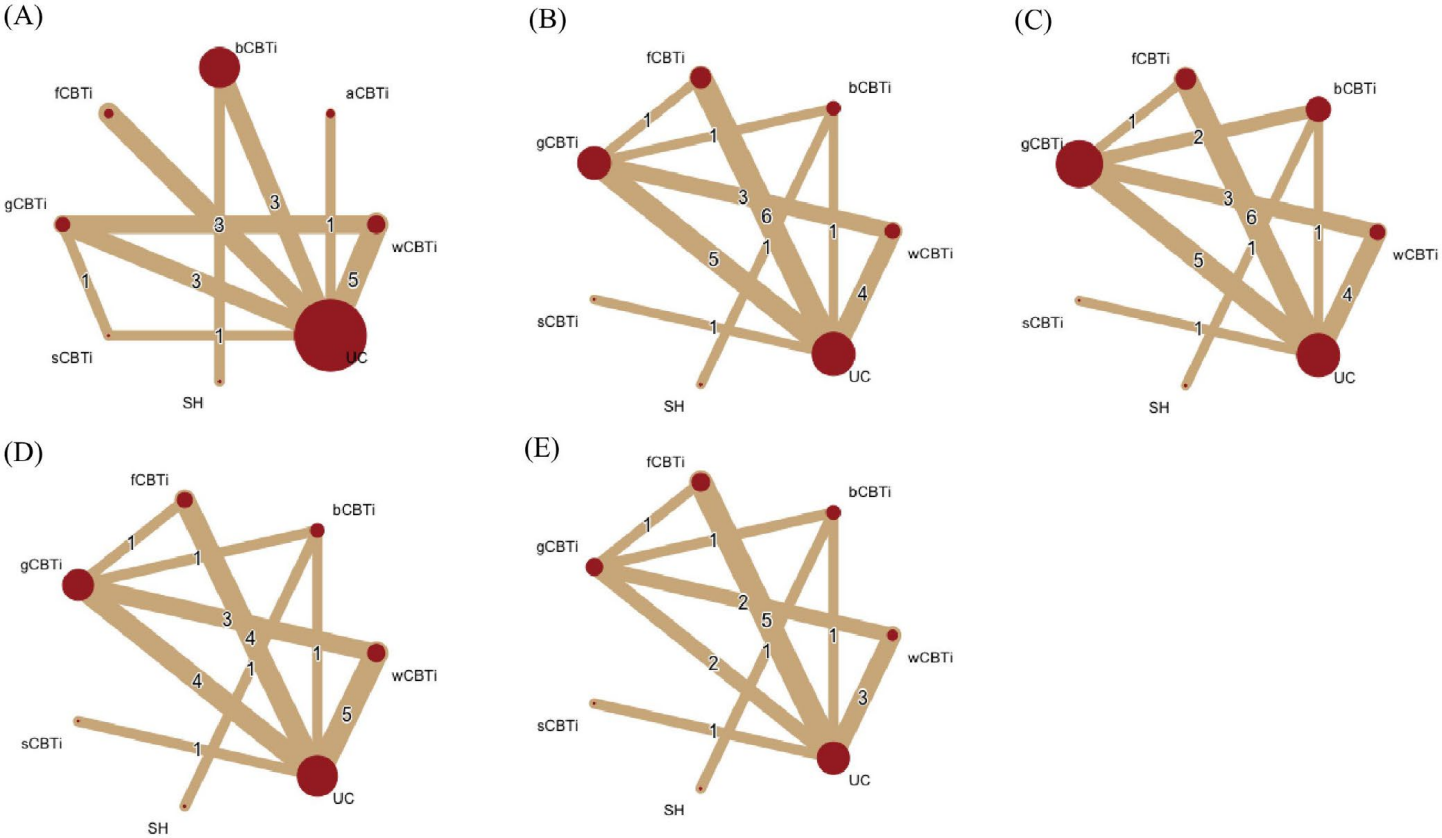
Network geometry for subjective sleep parameters in network meta‐analysis. (A) Insomnia severity; (B) total sleep time; (C) sleep onset latency; (D) sleep efficiency; (E) wake after sleep onset. The size of the nodes is related to the size of the population involved in each treatment. The thickness of the lines is proportional to the number of trials connected to the network. aCBTi, app‐based CBTi; bCBTi, brief CBTi; fCBTi, face‐to‐face cognitive behavioral therapy for insomnia; gCBTi, group CBTi; sCBTi, self‐help CBTi; SH, sleep hygiene; UC, usual care; wCBTi, web‐based CBTi.

Figure 2 presents network plots illustrating the direct comparisons among nonpharmacological interventions across the five sleep outcomes. The included studies formed a well‐connected network, with each plot comprising between one and seven treatment nodes. UC represented the largest sample size and is therefore depicted as the largest node in each plot. fCBTi was the intervention most frequently compared with UC, followed by wCBTi and gCBTi. The thickness of the connecting lines reflects the number of studies contributing to each treatment comparison.

### 3.3 Effects of Nonpharmacological Interventions on Sleep Outcomes

Table 3 presents the comparative effects of nonpharmacological interventions on sleep outcomes.

**TABLE 3:** Comparative effects of nonpharmacological interventions in improving sleep parameters.

Insomnia severity							
wCBTi				−0.12 (−0.85 to 0.62)			**−1.50 (−2.19 to −0.81)**
**−2.89 (−5.30 to −0.49)**	aCBTi						**−2.16 (−4.18 to −0.14)**
**−3.43 (−4.89 to −1.97)**	−0.54 (−2.66 to 1.59)	fCBTi					**−5.05 (−6.35 to −3.76)**
−3.24 (−6.65 to 0.18)	−0.34 (−4.09 to 3.41)	0.19 (−2.99 to 3.37)	sCBTi	1.00 (−2.33 to 4.33)			**−3.70 (−7.23 to −0.17)**
**−3.54 (−5.04 to −2.04)**	−0.64 (−2.80 to 1.51)	−0.11 (−0.81 to 0.59)	−0.30 (3.44 to 2.84)	gCBTi			**−1.43 (−2.26 to −0.59)**
**−4.14 (−5.84 to −2.45)**	−1.25 (−3.55 to 1.05)	−0.71 (−1.99 to 0.57)	−0.91 (−4.25 to 2.44)	−0.61 (−1.93 to 0.72)	bCBTi	−1.40 (−4.61 to 1.81)	−0.91 (−2.00 to 0.18)
**−5.54 (−9.18 to −1.91)**	−2.65 (−6.60 to 1.30)	−2.11 (−5.57 to 1.35)	−2.31 (−6.95 to 2.33)	−2.01 (−5.48 to 1.47)	−1.40 (−4.61 to 1.81)	SH	
**−5.05 (−6.35 to −3.76)**	**−2.16 (−4.18 to −0.14)**	**−1.62 (−2.29 to −0.96)**	−1.82 (−4.98 to 1.34)	**−1.52 (−2.27 to −0.76)**	−0.91 (−2.00 to 0.18)	0.49 (−2.91 to 3.89)	UC

Total sleep time (min)							
wCBTi			15.96 (−16.44 to 48.36)			7.56 (−16.65 to 31.77)	
−7.40 (−68.71 to 53.91)	bCBTi				−7.40 (−68.71 to 53.91)	22.40 (−12.83 to 57.63)	
26.02 (−8.61 to 60.65)	33.42 (−36.99 to 103.83)	fCBTi	−18.00 (−58.86 to 22.86)			9.91 (−6.30 to 26.12)	
27.91 (−3.85 to 59.66)	35.31 (−33.74 to 104.35)	1.89 (−19.58 to 23.35)	gCBTi		**38.27 (3.07 to 73.47)**	−1.78 (−22.25 to 18.69)	
32.80 (−14.60 to 80.19)	40.20 (−37.29 to 117.69)	6.78 (−31.57 to 45.12)	4.89 (−34.48 to 44.26)	sCBTi		0.00 (−35.17 to 35.17)	
32.52 (−4.56 to 69.61)	39.92 (−31.73 to 111.57)	6.50 (−19.51 to 32.52)	4.62 (−19.32 to 28.55)	−0.27 (−41.63 to 41.08)	SH		
**32.80 (1.03 to 64.56)**	40.20 (−28.85 to 109.25)	6.78 (−8.48 to 22.04)	4.89 (−12.80 to 22.58)	0.00 (−35.17 to 35.17)	0.27 (−21.47 to 22.02)	UC	

Sleep onset latency (min)							
wCBTi		7.08 (−22.78 to 36.93)	11.97 (−13.59 to 37.54)	**59.82 (17.38 to 102.26)**		13.11 (−10.82 to 37.05)	
−4.19 (−59.53 to 51.14)	sCBTi					3.40 (−40.90 to 47.70)	
9.81 (−18.15 to 37.76)	14.00 (−33.76 to 61.76)	bCBTi			14.00 (−33.76 to 61.76)	3.30 (−38.55 to 45.15)	
11.80 (−9.85 to 33.45)	15.99 (−42.63 to 74.62)	1.99 (−32.01 to 35.99)	gCBTi			14.46 (−5.50 to 34.42)	
16.74 (−5.60 to 39.07)	20.93 (−37.51 to 79.37)	6.93 (−26.74 to 40.60)	4.94 (−21.50 to 31.37)	fCBTi		14.66 (−3.93 to 33.25)	
19.73 (−27.80 to 67.26)	23.93 (−47.82 to 95.67)	9.93 (−43.61 to 63.46)	7.93 (−41.13 to 57.00)	3.00 (−44.55 to 50.54)	SH		
**23.13 (5.91 to 40.35)**	27.33 (−29.10 to 83.76)	13.33 (−16.73 to 43.38)	11.33 (−9.76 to 32.43)	6.40 (−10.86 to 23.65)	3.40 (−40.90 to 47.70)	UC	

Sleep efficiency (%)							
wCBTi				2.33 (−3.29 to 7.94)		7.99 (3.21 to 12.77)	
−0.85 (−16.22 to 14.51)	sCBTi					2.50 (−7.80 to 12.80)	
1.32 (−4.86 to 7.51)	2.18 (−13.42 to 17.78)	fCBTi		−1.56 (−10.90 to 7.78)		6.88 (1.41 to 12.34)	
1.55 (−7.39 to 10.48)	2.40 (−10.10 to 14.90)	0.22 (−9.11 to 9.56)	bCBTi	6.80 (−2.69 to 16.29)	2.40 (−10.10 to 14.90)	1.20 (−7.94 to 10.34)	
3.52 (−1.12 to 8.17)	4.38 (−10.64 to 19.39)	2.20 (−3.45 to 7.85)	1.98 (−6.34 to 10.30)	gCBTi		2.59 (−2.46 to 7.64)	
4.41 (−6.75 to 15.58)	5.27 (−12.91 to 23.44)	3.09 (−8.29 to 14.47)	2.87 (−10.32 to 16.06)	0.89 (−10.23 to 12.01)	SH		
**6.91 (2.60 to 11.23)**	7.77 (−7.20 to 22.74)	**5.59 (0.76 to 10.42)**	5.37 (−2.87 to 13.61)	3.39 (−0.80 to 7.58)	2.50 (−7.80 to 12.80)	UC	

Wake after sleep onset (min)							
wCBTi						−4.28 (−20.36 to 11.80)	
−0.50 (−41.30 to 40.30)	sCBTi					−7.70 (−33.43 to 18.03)	
−3.05 (−35.95 to 29.85)	−2.55 (−30.37 to 25.27)	fCBTi			0.62 (−22.58 to 23.82)	−6.16 (−17.56 to 5.25)	
−3.69 (−37.59 to 30.20)	−3.19 (−32.73 to 26.35)	−0.64 (−17.78 to 16.49)	gCBTi	−6.30 (−31.04 to 18.44)	−4.90 (−24.41 to 14.62)	−2.60 (−25.62 to 20.42)	
−3.30 (−26.80 to 20.20)	−2.80 (−36.15 to 30.55)	−0.25 (−23.28 to 22.78)	0.39 (−24.04 to 24.83)	bCBTi	−3.30 (−26.80 to 20.20)		
−6.58 (−38.64 to 25.47)	−6.08 (−35.23 to 23.07)	−3.53 (−18.57 to 11.51)	−2.89 (−18.73 to 12.95)	−3.28 (−25.09 to 18.52)	SH	−1.78 (−19.82 to 16.26)	
−8.20 (−39.86 to 23.46)	−7.70 (−33.43 to 18.03)	−5.15 (−15.73 to 5.43)	−4.51 (−19.01 to 9.99)	−4.90 (−26.12 to 16.32)	−1.62 (−15.30 to 12.06)	UC	

Certainty level	Efficacy						
	Good efficacy						
High							
Moderate							
Low							

*Note:* Values in bold indicate *p* < 0.05. The upper‐right cells present the results of direct head‐to‐head comparisons, and the lower‐left cells display results combining both direct and indirect comparisons. Mean differences are presented with 95% confidence intervals. A positive value indicates that participants receiving the treatment listed in the top row achieved a higher value for the corresponding sleep parameter than those receiving the treatment listed in the left‐hand column; a negative value indicates the opposite. Colour key: Blue = moderate certainty; dark grey = low certainty (CINeMA assessment). Orange diagonal cells indicate the interventions in the comparison matrix.

Abbreviations: aCBTi, app‐based CBTi; bCBTi, brief CBTi; fCBTi, face‐to‐face cognitive behavioral therapy for insomnia; gCBTi, group CBTi; sCBTi, self‐help CBTi; SH, sleep hygiene; UC, usual care; wCBTi, web‐based CBTi.

### 3.4 Insomnia Severity

wCBTi, aCBTi, fCBTi, and gCBTi were associated with significantly lower insomnia severity than UC, with MDs of −5.05, −2.16, −1.62, and −1.52, respectively. wCBTi also resulted in a significantly greater reduction in insomnia severity compared with aCBTi, fCBTi, sCBTi, gCBTi, bCBTi, and SH. The P score indicated that wCBTi was most likely (99%) to be the optimal treatment for reducing insomnia severity (Table 4).

**TABLE 4:** P score for treatment ranking.

Rank	Insomnia	*p*‐score	TST	*p*‐score	SOL	*p*‐score
	Treatments		Treatments		Treatments	
1	wCBTi	0.99	wCBTi	0.86	wCBTi	0.79
2	aCBTi	0.68	bCBTi	0.81	sCBTi	0.72
3	fCBTi	0.59	fCBTi	0.49	bCBTi	0.53
4	sCBTi	0.57	gCBTi	0.43	gCBTi	0.50
5	gCBTi	0.54	sCBTi	0.33	fCBTi	0.39
6	bCBTi	0.36	SH	0.31	SH	0.37
7	SH	0.16	UC	0.27	UC	0.20
8	UC	0.12				

Rank	SE	*p*‐score	WASO	*p*‐score
	Treatments		Treatments	
1	wCBTi	0.74	wCBTi	0.60
2	sCBTi	0.68	sCBTi	0.59
3	fCBTi	0.62	fCBTi	0.57
4	bCBTi	0.57	gCBTi	0.53
5	gCBTi	0.40	bCBTi	0.52
6	SH	0.38	SH	0.39
7	UC	0.11	UC	0.29

Abbreviations: aCBTi, app‐based CBTi; bCBTi, brief CBTi; fCBTi, face‐to‐face cognitive behavioral therapy for insomnia; gCBTi, group CBTi; sCBTi, self‐help CBTi; SE, sleep efficiency; SH, sleep hygiene; SOL, sleep onset latency; TST, total sleep time; UC, usual care; WASO, wake after sleep onset; wCBTi, web‐based CBTi.

### 3.5 TST

wCBTi was associated with significantly longer TST than UC (MD = 32.80 min). The P score revealed that wCBTi was most likely (86%) to be the optimal treatment for improving TST (Table 4).

### 3.6 SOL

wCBTi was associated with significantly shorter sleep onset latency compared with UC (MD = −23.13 min). The P score suggested that wCBTi (79%) was most likely to be the optimal treatment for reducing SOL (Table 4).

### 3.7 SE

wCBTi and fCBTi were associated with significantly higher SE than UC (MDs = 6.91% and 5.59%, respectively). The P score indicated that wCBTi (74%) was most likely to be the best treatment for improving SE (Table 4).

### 3.8 WASO

No treatment demonstrated superior efficacy to any other.

### 3.9 Inconsistency Testing

The design‐by‐treatment and side‐splitting inconsistency models exhibited no inconsistencies across the five sleep outcomes (Table S4).

### 3.10 Risk of Bias Assessment

The findings of the Cochrane RoB 2.0 assessment revealed that 68% of the included studies were judged to have a low risk of bias across all domains and that 32% were judged to have some concerns. These concerns primarily arose from problems related to the randomization process, deviations from intended interventions, and selective reporting of results. Several studies did not provide sufficient details on the availability of multiple eligible outcome measures or the conduct of multiple eligible analyses (Figure S1).

### 3.11 Publication Bias

No evidence of publication bias was observed for insomnia severity, TST, SOL, SE, or WASO outcomes (Table S5).

### 3.12 Heterogeneity

Quantitative heterogeneity varied across outcomes. The network heterogeneity estimates were high for all outcomes, with τ^2^ = 321.96 and *I* ^2^ = 99.4% (95% CI: 99.3%–99.5%) for TST, τ^2^ = 455.95 and *I* ^2^ = 97.1% (95% CI: 96.3%–97.8%) for SOL, τ^2^ = 21.74 and *I* ^2^ = 89.1% (95% CI: 83.1%–92.9%) for SE, τ^2^ = 137.89 and *I* ^2^ = 87.5% (95% CI: 79.0%–92.5%) for WASO, and τ^2^ = 0.38 and *I* ^2^ = 79.6% (95% CI: 65.9%–87.8%) for insomnia severity. These findings indicate substantial between‐study heterogeneity, particularly for total sleep time and sleep onset latency.

### 3.13 Confidence in Evidence

The certainty of evidence for the sleep outcomes, as evaluated using the CINeMA tool based on the GRADE framework, ranged from high to low. For insomnia severity, 22, 5, and 1 of 28 comparisons received high, moderate, and low confidence ratings, respectively. For TST, 17, 3, and 1 of 21 comparisons were rated as high, moderate, and low confidence, respectively. For SOL, 16, 4, and 1 comparisons were rated as high, moderate, and low, respectively. For SE, 15, 5, and 1 comparisons were rated as high, moderate, and low, respectively. For WASO, 14, 5, and 2 comparisons received high, moderate, and low ratings, respectively. Further details are provided in Table 3.

### 3.14 Meta‐Regression Analysis

Meta‐regression analysis showed that treatment intensity, defined as total intervention minutes, was not significantly associated with treatment effects across sleep outcomes (*p* > 0.05). Similarly, study quality (low risk of bias vs. some concerns) and inclusion of all four core CBTi components (included vs. not included) did not significantly influence the observed treatment effects (*p* > 0.05).

## 4 Discussion

To the best of our knowledge, this NMA is the first to systematically evaluate and compare different CBTi delivery formats to identify the most effective intervention for adolescent insomnia, a condition that has substantial effects on health and development in this population (Short et al. 2013; Owens and Weiss 2017; Johnson et al. 2006). The findings demonstrated that wCBTi was particularly effective in improving key sleep parameters, including insomnia severity, TST, SOL, and SE, significantly outperforming UC. This result is consistent with that reported by Hasan et al. (2022) who observed similar benefits of wCBTi in adults, supporting its applicability across age groups. Unlike traditional pairwise meta‐analyses that provide only direct, binary comparisons, NMA allows a comprehensive ranking of CBTi modalities, highlighting the superiority of wCBTi over other approaches. These findings provide clear guidance for clinicians in selecting effective interventions for adolescent insomnia.

The magnitude of the observed effects suggests that wCBTi may provide clinically meaningful benefits for adolescents with insomnia. Previous insomnia research has suggested minimal important differences of approximately 10 min for SOL and 5% for sleep efficiency, while changes in ISI scores have commonly been interpreted using thresholds of approximately 2.6 to 6 points, depending on whether between‐group or within‐person change is considered (Bastien et al. 2001; Yang et al. 2009; Edinger et al. 2021). In the present network meta‐analysis, wCBTi reduced SOL by approximately 23 min, improved sleep efficiency by approximately 7%, and reduced insomnia severity by approximately 5 points. These findings suggest that the benefits of wCBTi are likely to be clinically relevant rather than merely statistically significant. In addition to its clinical efficacy, wCBTi demonstrates considerable potential because of its scalability, cost‐effectiveness, and flexibility in delivery (Drake 2016). These characteristics make it particularly suitable for adolescents, who are generally more receptive to technology‐based interventions and may value the privacy and autonomy provided by web‐based formats (Ye et al. 2015). Moreover, wCBTi can be incorporated into school‐based health programs or primary care settings, thereby improving early access to intervention. Ongoing innovations in digital platforms, such as AI‐driven personalization and interactive engagement tools, may further enhance user participation and therapeutic outcomes (Espie et al. 2012). Continued research is warranted to refine the structure, timing, and support components of wCBTi to optimize its effectiveness and promote equitable access among diverse adolescent populations.

Compared with other CBTi delivery formats, wCBTi provides a balanced approach that combines structured therapeutic content with flexible and accessible delivery. fCBTi is effective but can pose logistical barriers such as scheduling conflicts, geographic limitations, and the stigma associated with attending therapy sessions, factors that may deter adolescents from participation (Taylor et al. 2017). aCBTi is convenient but lacks the therapist interaction and feedback that many adolescents require to sustain motivation and behavioral change (Gumport et al. 2024). gCBTi can lead to discomfort resulting from social anxiety or fear of self‐disclosure among peers. By contrast, wCBTi integrates personalized guidance while maintaining privacy and user control, features essential for adolescent engagement. This combination of therapeutic integrity and contextual adaptability likely explains the consistently superior outcomes of wCBTi observed in this NMA.

### 4.1 Limitations

This analysis has several limitations. First, substantial heterogeneity among the included studies resulting from variations in intervention protocols, participant characteristics, outcome measures, and methodological rigor may have affected the internal validity of the findings. Second, the uneven distribution of studies across CBTi modalities and intervention components limited statistical power for some comparisons and may have reduced the precision and robustness of the estimated effects. Third, no delivery format demonstrated superiority for WASO. This result aligns with research indicating that WASO is typically less responsive to behavioral interventions than sleep initiation outcomes (Ye et al. 2015; Hasan et al. 2022). Several factors may account for this observation. CBTi components primarily target difficulties with sleep initiation rather than sleep maintenance (Trauer et al. 2015; Espie et al. 2012). Additionally, self‐reported WASO is subject to greater measurement variability than objective assessments such as actigraphy (Bei et al. 2014; Ye et al. 2015). Environmental or comorbid factors, such as noise, cosleeping, and psychiatric symptoms may also contribute to persistent WASO (Taylor et al. 2017; de Zambotti et al. 2018; Gumport et al. 2024). Future studies should develop targeted interventions to reduce WASO, apply standardized CBTi protocols, and include objective sleep measures to enhance accuracy and generalizability. Fourth, the absence of direct head‐to‐head trials comparing face‐to‐face CBTi with web‐based CBTi limits the certainty of conclusions regarding their relative effectiveness.

## 5 Linking Evidence to Action

- Prioritize web‐based CBT‐i as an accessible first‐line behavioral treatment option for adolescents with insomnia, using a stepped‐care approach to refer complex or nonresponsive cases to therapist‐led CBT‐i.
- Integrate insomnia screening and brief sleep assessments into school health services, primary care, and adolescent nursing practice to support early identification and timely intervention.
- Embed web‐based CBT‐i within school‐based or primary‐care pathways, with attention to equity, device and internet access, privacy, and age‐appropriate engagement features to support adherence.
- Provide nursing‐led sleep education for adolescents and families, including sleep hygiene, stimulus control, sleep restriction principles, and guidance on when to seek specialist care.
- Evaluate CBT‐i implementation in real‐world settings through pragmatic studies that assess adherence, long‐term effectiveness, cost‐effectiveness, and objective sleep outcomes when feasible.

## 6 Conclusions

This NMA provides robust evidence supporting wCBTi as the optimal delivery format for improving sleep outcomes among adolescents. The demonstrated efficacy, accessibility, and adaptability of wCBTi position wCBTi as a valuable first‐line treatment particularly suitable for digital‐native adolescent populations. Greater integration of wCBTi interventions into clinical practice could substantially enhance insomnia management and result in improved long‐term health and developmental outcomes in adolescents.

Future large‐scale, high‐quality RCTs with standardized intervention protocols and consistent outcome assessments are required to validate and expand these findings, particularly through direct head‐to‐head comparisons between fCBTi and wCBTi to clarify their relative efficacy, acceptability, scalability, and implementation potential in adolescent populations. Further research on the long‐term sustainability of treatment effects, particularly within web‐based interventions, is critical for establishing effective insomnia management strategies for adolescents. In addition, future dismantling trials and component network meta‐analyses are needed to clarify the independent and combined effects of CBTi delivery format, treatment components, and intervention intensity among adolescents with insomnia.

## Funding

This study was supported by grants awarded to H.Y.C. from the National Science and Technology Council of Taiwan (NSTC 114‐2314‐B‐038‐094‐MY3 and NSTC 113‐2628‐B‐038‐003‐MY3).

## Disclosure

Statement on Textual Overlap: The authors acknowledge limited textual overlap with previously published work by the same research group, primarily in descriptions of standard methodological procedures and general background concepts common to systematic reviews and network meta‐analyses. All overlapping text has been revised and rephrased to ensure originality, and relevant prior publications are appropriately cited. The present study addresses a distinct research question, population, and intervention context and represents an independent and original contribution.

## Conflicts of Interest

The authors declare no conflicts of interest.

## Supporting information


**Table S1:** Example of searching.
**Table S2:** List of the excluded studies after a full‐text review.
**Table S3:** Outcome measurement of self‐reported insomnia symptoms.
**Table S4:** Design‐by‐treatment inconsistency.
**Table S5:** Publication bias of included studies.
**Figure S1:** Risk of bias (RoB 2.0).

## Data Availability

The data that support the findings of this study are available from the corresponding author upon reasonable request.
