# Supplementary material for: Comparative Effects of Various Modalities of Cognitive Behavioral Therapy for Insomnia in Adolescents: A Systematic Review and Network Meta‐Analysis
Source: Worldviews Evid Based Nurs. 2026 Jul 13;23(4):e70158. doi: 10.1111/wvn.70158 (PMC13360328; doi:10.1111/wvn.70158)
Supplement: Supplementary file 1 — Table S1: Example of searching. Table S2: List of the excluded studies after a full‐text review. Table S3: Outcome measurement of self‐reported insomnia symptoms. Table S4: Design‐by‐treatment inconsistency. Table S5: Publication bias of included studies. Figure S1: Risk of bias (RoB 2.0). [file WVN-23-0-s001.docx]

**Supplementary Online Content**

Table S1. Example of searching

Table S2. List of the excluded studies after a full-text review

Table S3. Outcome measurement of self-reported insomnia symptoms

Table S4. Design-by-treatment inconsistency

Table S5. Publication bias of included studies

Figure S1. Risk of bias (RoB 2.0)

Table S1. Example of searching

| **Database** | **Line** | **Concept** | **Search terms** |
| --- | --- | --- | --- |
| **PubMed** | #1 | Insomnia/sleep terms | (insomnia[MeSH Terms] OR "Sleep Initiation and Maintenance Disorders"[MeSH Terms] OR insomnia*[Title/Abstract] OR "sleep deprivation"[Title/Abstract] OR "daytime sleepiness"[Title/Abstract] OR "sleep disturbance*"[Title/Abstract] OR "sleep disorder*"[Title/Abstract] OR "sleep quality"[Title/Abstract]) |
|  | #2 | CBT-i/intervention terms | ("Cognitive Behavioral Therapy"[MeSH Terms] OR "cognitive behavioral therap*"[Title/Abstract] OR "cognitive behaviour therap*"[Title/Abstract] OR CBT-I[Title/Abstract] OR CBTi[Title/Abstract] OR "cognitive behavioral therapy for insomnia"[Title/Abstract] OR "cognitive behaviour therapy for insomnia"[Title/Abstract] OR "sleep hygiene"[Title/Abstract] OR "stimulus control"[Title/Abstract] OR "sleep restriction"[Title/Abstract] OR "relaxation therapy"[Title/Abstract] OR "brief therap*"[Title/Abstract] OR "internet-based"[Title/Abstract] OR "web-based"[Title/Abstract] OR "group-based"[Title/Abstract]) |
|  | #3 | Age group | (adolescent[MeSH Terms] OR child[MeSH Terms] OR adolescen*[Title/Abstract] OR teenager*[Title/Abstract] OR youth[Title/Abstract] OR child*[Title/Abstract]) |
|  | #4 | Study design | (randomized controlled trial[Publication Type] OR controlled clinical trial[Publication Type] OR random*[Title/Abstract] OR trial[Title/Abstract]) |
|  | **#5** | **Final search** | **#1 AND #2 AND #3 AND #4** |
| **Embase** | #1 | Insomnia/sleep terms | ('insomnia'/exp OR 'sleep initiation and maintenance disorder'/exp OR insomnia*:ti,ab OR 'sleep deprivation':ti,ab OR 'daytime sleepiness':ti,ab OR 'sleep disturbance*':ti,ab OR 'sleep disorder*':ti,ab OR 'sleep quality':ti,ab) |
|  | #2 | CBT-i/intervention terms | ('cognitive behavioral therapy'/exp OR 'cognitive behavior therapy':ti,ab OR 'cognitive behavioural therapy':ti,ab OR CBT-I:ti,ab OR CBTi:ti,ab OR 'cognitive behavioral therapy for insomnia':ti,ab OR 'cognitive behaviour therapy for insomnia':ti,ab OR 'sleep hygiene':ti,ab OR 'stimulus control':ti,ab OR 'sleep restriction':ti,ab OR 'relaxation therapy':ti,ab OR 'brief therap*':ti,ab OR 'internet-based':ti,ab OR 'web-based':ti,ab OR 'group-based':ti,ab) |
|  | #3 | Age group | ('adolescent'/exp OR 'child'/exp OR adolescen*:ti,ab OR teenager*:ti,ab OR youth:ti,ab OR child*:ti,ab) |
|  | #4 | Study design | ('randomized controlled trial'/de OR random*:ti,ab OR trial:ti,ab) |
|  | **#5** | **Final search** | **#1 AND #2 AND #3 AND #4** |
| **PsycINFO** | #1 | Insomnia/sleep terms | (DE "Insomnia" OR DE "Sleep Disorders" OR insomnia* OR "sleep deprivation" OR "daytime sleepiness" OR "sleep disturbance*" OR "sleep disorder*" OR "sleep quality") |
|  | #2 | CBT-i/intervention terms | (DE "Cognitive Behavior Therapy" OR "cognitive behavioral therap*" OR "cognitive behaviour therap*" OR CBT-I OR CBTi OR "cognitive behavioral therapy for insomnia" OR "cognitive behaviour therapy for insomnia" OR "sleep hygiene" OR "stimulus control" OR "sleep restriction" OR "relaxation therapy" OR "brief therap*" OR "internet-based" OR "web-based" OR "group-based") |
|  | #3 | Age group | (DE "Adolescence" OR DE "Childhood" OR adolescen* OR teenager* OR youth OR child*) |
|  | #4 | Study design | (random* OR trial OR "controlled trial") |
|  | **#5** | **Final search** | **#1 AND #2 AND #3 AND #4** |
| **CINAHL** | #1 | Insomnia/sleep terms | (MH "Insomnia+" OR MH "Sleep Disorders+" OR insomnia* OR "sleep deprivation" OR "daytime sleepiness" OR "sleep disturbance*" OR "sleep disorder*" OR "sleep quality") |
|  | #2 | CBT-i/intervention terms | (MH "Cognitive Therapy+" OR "cognitive behavioral therap*" OR "cognitive behaviour therap*" OR CBT-I OR CBTi OR "cognitive behavioral therapy for insomnia" OR "cognitive behaviour therapy for insomnia" OR "sleep hygiene" OR "stimulus control" OR "sleep restriction" OR "relaxation therapy" OR "brief therap*" OR "internet-based" OR "web-based" OR "group-based") |
|  | #3 | Age group | (MH "Adolescence+" OR MH "Child+" OR adolescen* OR teenager* OR youth OR child*) |
|  | #4 | Study design | (MH "Randomized Controlled Trials" OR random* OR trial OR "controlled trial") |
|  | **#5** | **Final search** | **#1 AND #2 AND #3 AND #4** |
| **Cochrane Library** | #1 | Insomnia/sleep terms | ([mh insomnia] OR [mh "Sleep Initiation and Maintenance Disorders"] OR insomnia*:ti,ab,kw OR "sleep deprivation":ti,ab,kw OR "daytime sleepiness":ti,ab,kw OR "sleep disturbance*":ti,ab,kw OR "sleep disorder*":ti,ab,kw OR "sleep quality":ti,ab,kw) |
|  | #2 | CBT-i/intervention terms | ([mh "Cognitive Behavioral Therapy"] OR "cognitive behavioral therap*":ti,ab,kw OR "cognitive behaviour therap*":ti,ab,kw OR CBT-I:ti,ab,kw OR CBTi:ti,ab,kw OR "cognitive behavioral therapy for insomnia":ti,ab,kw OR "cognitive behaviour therapy for insomnia":ti,ab,kw OR "sleep hygiene":ti,ab,kw OR "stimulus control":ti,ab,kw OR "sleep restriction":ti,ab,kw OR "relaxation therapy":ti,ab,kw OR "brief therap*":ti,ab,kw OR "internet-based":ti,ab,kw OR "web-based":ti,ab,kw OR "group-based":ti,ab,kw) |
|  | #3 | Age group | ([mh adolescent] OR [mh child] OR adolescen*:ti,ab,kw OR teenager*:ti,ab,kw OR youth:ti,ab,kw OR child*:ti,ab,kw) |
|  | #4 | Study design | (random*:ti,ab,kw OR trial:ti,ab,kw OR "controlled trial":ti,ab,kw) |
|  | **#5** | **Final search** | **#1 AND #2 AND #3 AND #4** |

**Table S2. List of the excluded studies after a full-text review**

|  | Reasons of exclusion |
| --- | --- |
| Blake et all 2016 (1) | Insufficient data for analyses even after contacted the author |
| Blake et all 2017 (2) | Insufficient data for analyses even after contacted the author |
| Weiss et al 2006 (3) | Insufficient data for analyses even after contacted the author |

References

1. Blake, M., Waloszek, J. M., Schwartz, O., Raniti, M., Simmons, J. G., Blake, L., Murray, G., Dahl, R. E., Bootzin, R., Dudgeon, P., Trinder, J., & Allen, N. B. (2016). The SENSE study: Post intervention effects of a randomized controlled trial of a cognitive–behavioral and mindfulness-based group sleep improvement intervention among at-risk adolescents. Journal of Consulting and Clinical Psychology, 84(12), 1039–1051. <https://doi.org/10.1037/ccp0000142>
2. Matthew Blake, Orli Schwartz, Joanna M. Waloszek, Monika Raniti, Julian G. Simmons, Greg Murray, Laura Blake, Ronald E. Dahl, Richard Bootzin, Dana L. McMakin, Paul Dudgeon, John Trinder, Nicholas B. Allen, The SENSE Study: Treatment Mechanisms of a Cognitive Behavioral and Mindfulness-Based Group Sleep Improvement Intervention for At-Risk Adolescents, Sleep, Volume 40, Issue 6, June 2017, zsx061, <https://doi.org/10.1093/sleep/zsx061>
3. Weiss MD, Wasdell MB, Bomben MM, Rea KJ, Freeman RD. Sleep hygiene and melatonin treatment for children and adolescents with ADHD and initial insomnia. J Am Acad Child Adolesc Psychiatry. 2006;45(5):512-519.

**Table S3. Outcome measurement of self-reported insomnia symptom**

| - The 7-items insomnia severity index [ISI]; [1] each item scale, 1-4; range 0-28; higher score indicating more severe insomnia. - The 32-items Holland sleep disorder questionnaire [HSDQ]; [2] consisting of 8-items insomnia subscale; each item scale 1-5; range: 0-40; higher score indicating more severe sleep problem. |
| --- |

Reference:

[1] Bastien CH, Vallières A, Morin CM. Validation of the Insomnia Severity Index as an outcome measure for insomnia research. *Sleep Med.* 2001;2(4):297-307.

[2] Kerkhof GA, Geuke ME, Brouwer A, Rijsman RM, Schimsheimer RJ, Van Kasteel V. Holland Sleep Disorders Questionnaire: a new sleep disorders questionnaire based on the International Classification of Sleep Disorders‐2. *J Sleep Res.* 2013;22(1):104-107.

**Table S4. Design-by-treatment inconsistency**

| **Sleep parameter** | **Q** | **P value** |
| --- | --- | --- |
| Total sleep time | 4.55 | 0.47 |
| Sleep onset latency | 5.76 | 0.45 |
| Wake after sleep onset | 0.90 | 0.93 |
| Sleep efficiency | 5.55 | 0.35 |
| Insomnia severity | 7.06 | 0.07 |

**Table S5. Publication bias of included studies**

| **Sleep outcomes** | **P value** |
| --- | --- |
| Total sleep time | 0.38 |
| Sleep onset latency | 0.20 |
| Wake up after sleep onset | 0.58 |
| Sleep efficiency | 0.72 |
| Insomnia severity | 0.54 |

Figure S1. Risk of bias (RoB 2.0)
